# Advances in Solidification Processing in Steady Magnetic Field

**DOI:** 10.3390/ma18122886

**Published:** 2025-06-18

**Authors:** Shengya He, Chenglin Huang, Chuanjun Li

**Affiliations:** 1 GRINM (Guangdong) Institute for Advanced Materials and Technology, Foshan 528051, China; 2 School of Materials Science and Engineering, Shanghai University, Shanghai 200444, China; cl_huang@shu.edu.cn; 3 School of Iron and Steel, Soochow University, Suzhou 215031, China

**Keywords:** metal solidification, steady magnetic field, undercooling, interfacial energy, microsegregation

## Abstract

As a contactless physical field, a steady magnetic field (SMF) is capable of acting on substances, which leads to changes in physical and/or chemical properties and to further influencing thermodynamic and kinetic behaviors at macroscopic, mesoscopic, and microscopic scales. The application of the SMF to material science has evolved into an important interdisciplinary field—the Electromagnetic Processing of Materials (EPM). Therein, the implementation of the SMF for the solidification of metals and alloys has been increasingly given attention. The SMF was found to regulate nucleation, crystal growth, the distribution of solutes and structure morphology during alloy solidification via various magnetic effects, such as magnetic damping, the thermoelectric magnetic effect, magnetic orientation and magnetically controlled diffusion. In this review, we briefly summarize the main SMF effects and review recent progress in magnetic field-assisted solidification processing, including nucleation, dendritic growth, solute segregation and interfacial phenomena. Finally, future perspectives regarding controlling alloys’ solidification using an SMF are discussed.

## 1. Introduction

The steady magnetic field (SMF), characterized by its non-contact interaction and multiple magnetic effects, has been extensively utilized in materials processing, thereby developing an emerging research field known as the Electromagnetic Processing of Materials (EPM). Over the past decades, advancements in strong magnet technology have enabled laboratories to generate an SMF up to ~40 T, significantly advancing materials science in the SMF [1]. Extensive research has demonstrated that the application of an SMF profoundly influences metal solidification, including nucleation temperature [2,3,4], orientation [5,6,7], solid-state diffusion [8,9], melt convection [10,11] and dendritic growth [12,13]. Leveraging these effects, material scientists aim to regulate alloys’ solidification using an SMF to improve casting quality. Therefore, on the one hand, scientists make efforts to reveal the physical mechanisms of these magnetic phenomena. On the other hand, they try to explore the possibility of an engineering application. Up to now, a series of technologies for high-quality castings assisted by the SMF has been established and well-applied based on its contamination-free characteristics and various magnetic effects. The main metallurgical processes and material processes are covered, even including bulk crystal growth in semiconductor manufacture. For example, the SMF is used to reduce the impact depth of liquid steel from the nozzle in the crystallizer, to stabilize the liquid level and to avoid slag entrapment during continuous casting. The solute homogeneity and microstructure of heat-treated casting ingots can be adjusted by magnetic field-controlled diffusion technology. The effect of the SMF on the fluid flow has been exploited to eliminate defection in high-quality bulk crystal relating to semiconductors. The application of the SMF becomes one of the important methods that improve the macro/micro structures and performance of materials. In consideration of many new research results over the past decades, it is time to give an overview of these research works. This review summarizes recent advances in alloy solidification within an SMF and offers useful guidance for researchers working in this area.

## 2. Main Magnetic Effects During Solidification

As an essential physical parameter comparable to pressure and temperature, the magnetic field possesses distinct advantages, including multiple magnetic effects, contactless interaction and strong directionality. Since any substance has magnetism, such as ferromagnetism, paramagnetism and diamagnetism [14], the action of the SMF on the substance may result in the emergence of novel phenomena, even some great discoveries. It is evidence of this that nineteen Nobel prizes over the past century are related to magnetic phenomena. In the following section, the main magnetic effects relating to solidification are summarized, including magnetic damping (MD), thermoelectric magnetic convection (TEMC), magnetic orientation and diffusion.

### 2.1. Magnetic Damping

In the field of EPM, one of the fundamental theoretical frameworks is magnetohydrodynamics (MHD). It is well known that the essence of alloy solidification is the liquid-to-solid phase transformation. The heat and mass transfer in the liquid could be influenced by the fluid flow. Since the SMF can alter the flow behavior of conductive melts, the SMF can tailor dendritic growth, solute distribution, interface morphology and solidification structures. The influence of the SMF on fluid flow primarily manifests itself in two aspects: MD and TEMC.

**Figure 1:**
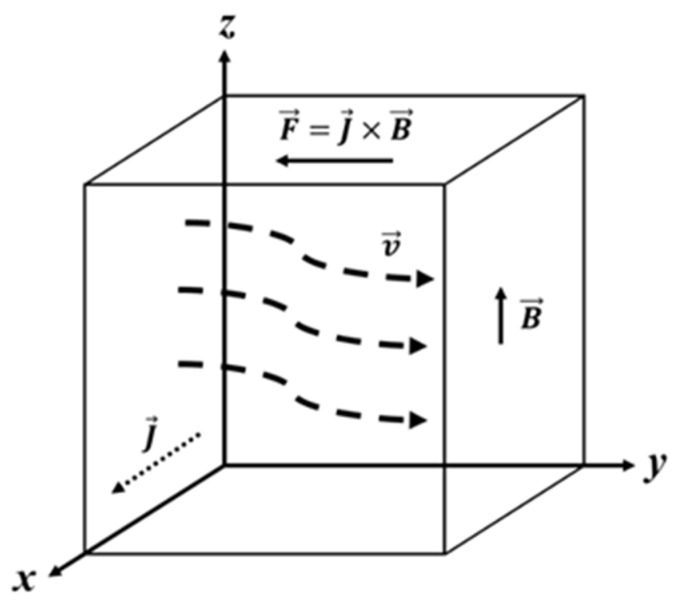
Illustrative sketch of the MD.

The suppression of conductive liquid flow by the SMF is known as the MD. The MD effect derives from Faraday’s law of electromagnetic induction. The interaction between the induction current and the SMF will generate Lorentz force. The direction of this force is in the opposite direction to the fluid flow. As a result, this force can effectively reduce the fluid velocity. When the magnetic field intensity reaches a certain value, the flow has a possibility of being suppressed completely [11]. The governing equation for fluid motion in the SMF can be expressed as [15]

(1) $$\rho \frac{d\vec{v}}{dt}=\rho \vec{F}-\nabla p+\eta \nabla^{2}\vec{v}+\vec{J}\times \vec{B}$$

where ρ is density, $\vec{J}$ is inducing current, p is pressure, η is dynamic viscosity, $\vec{B}$ is magnetic intensity, $\vec{v}$ is fluid velocity, and $\vec{F}$ is volume force. The principal schematic of MD is shown in Figure 1. Oreper et al. [16] pointed out that the effectiveness of the MD is primarily determined by magnetic field intensity, characteristic length scales and the geometry of the container, making it difficult to completely suppress fluid flow. The MD on melt flow has been widely utilized during solidification to improve microstructures and properties. For instance, the MD has successfully been applied to continuous casting and single-crystal silicon growth. The SMF devices are installed around the mold, and the electromagnetic force effectively suppresses turbulent flow. This leads to a reduction in mold level fluctuations, slag entrapment and the impingement of molten steel on the solidifying shell and thereby to the better quality of the cast billet [17,18,19]. Similarly, for single-crystal silicon growth, an SMF is always employed to suppress melt convection, mitigate thermal fluctuations and minimize the formation of unwanted grains and banded segregation [20,21]. Furthermore, the measurement of liquid-phase diffusion coefficients on Earth is often conducted in an SMF, because the liquid-phase diffusion is always affected by natural convection induced by gravity, leading to inaccuracies. Frohberg et al. [22] found that the diffusion coefficient of In-Sn melts follows an exponential law in microgravity, whereas on Earth, it adheres to the Arrhenius law. Utilizing the MD to eliminate natural convection in terrestrial environments has emerged as a novel approach for obtaining precise liquid-phase diffusion coefficients. Cahoon and Youdelis [23] first quantified the liquid-phase diffusion coefficient of the Bi-Sb system in a 3.4 T SMF, reporting that while the magnetic field significantly reduced convective velocity, the diffusion of Sb atoms in the Bi melt remained unaffected by changes in magnetic field intensity. Since then, numerous investigations of the liquid-phase diffusion behavior in alloy systems in the SMF have been performed [24,25,26].

### 2.2. Thermoelectric Magnetic Convection

At the end of the 20th century, some alloy solidification phenomena in the SMF could not be explained by the MD or the magnetization force. Tewari et al. [27] revealed that the cellular structures in the mushy zone were distorted severely in directionally solidified Pb-Sn alloy within a 0.45 T transverse magnetic field. Similarly, Lehmann et al. [28] applied a vertical magnetic field during the horizontal directional solidification of Cu-Ag and Al-Cu alloys and observed the change in dendritic morphology as the magnetic field intensity increased. They found that higher magnetic field intensities led to the gradual evolution of dendrites into irregular structures, accompanied by the formation of spotted patterns. The dendritic morphology still remained at the condition of exceeding the magnetic field threshold. The primary dendritic arm spacing of Cu-Ag alloy exhibited an increase, followed by a decrease, whereas the primary dendrite spacing of Al-Cu alloy rose monotonically with an increasing magnetic field intensity.

**Figure 2:**
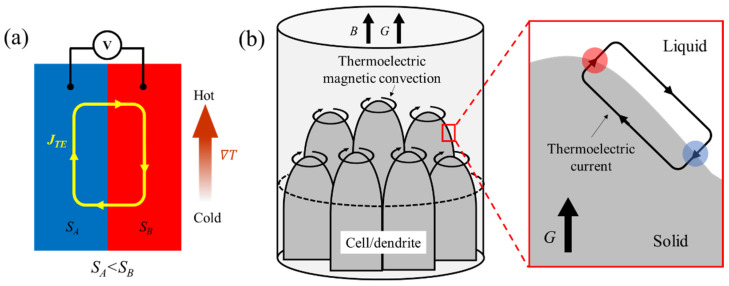
(**a**) Schematic illustration of the Seebeck effect. (**b**) The formation of TEMC within the mushy zone.

These studies indicated the existence of a novel type of convection in solidified alloys in the SMF, known as TEMC. It is widely reported that the TEMC arises from the coupling effect in between thermoelectric current (TEC) and magnetic fields. The TEC is generated owing to the Seebeck effect at the solid/liquid interface. The Seebeck effect refers to the generation of a voltage difference in two different conductive materials caused by a temperature gradient. When the thermoelectric power difference ∇*S* is not parallel to the temperature gradient ∇*T*, a TEC will form. Figure 2 illustrates the principle of the Seebeck effect and the formation of TEMC. When the SMF is applied, the TEC interacts with it to generate a Lorentz force, namely thermoelectric magnetic force (TEMF) [29]. This force simultaneously applies to the solidified area and the melt. The latter gives rise to the appearance of TEMC, the concept of which was first introduced by Shercliff [10] when he investigated the MHD of liquid lithium cooling systems in magnetically confined nuclear reactors. Later, Gel’fgat and Gorbunnov [30] observed the significant deformation of In-Sb crystals during directional growth in an SMF, providing the first experimental evidence of TEMC during crystal growth. Subsequent intensive studies further validated the TEMC effect during solidification. Moreau et al. [12] found that the TEMC induced by a 0.55 T magnetic field is beneficial to dendritic coarsening in directionally solidified Bi-Sn alloys. Khine et al. [31] conducted a numerical simulation of TEMC during the Bridgman growth of semiconductor crystals and found that with the magnetic field intensity increasing from zero, the meridional circulation initially rose to a maximum at a Hartmann number of approximately ten and gradually diminished toward zero as the MD effect began to dominate over the TEMC.

During solidification, the above-mentioned two magnetic effects act simultaneously on the alloy melt and alter heat and mass transfer within the melt. The two magnetic effects have opposing influences on melt flow. MD suppresses fluid motion. In contrast, TEMC promotes melt convection. It is indicated that there is a force balance between TEMF, inertial force and MD force, while the frictional forces can be neglected at the macroscale [32]. At the microscale, however, the Reynolds number is far less than 1, which indicates that the viscous friction dominates over inertia force and balances with the TEMF and MD force. Based on mechanical equilibrium conditions, the relationship between melt velocity (*u*) and magnetic field intensity (*B*) at both macro- and microscales can be derived.

At the macroscopic scale, the balance conditions for different magnetic field intensities are as follows:

In a weak SMF, the TEMF balances the inertial force, i.e., $\rho u_{1}^{2}/\lambda \approx \sigma \mathrm{SGB}$. It gives the corresponding melt velocity:

(2) $$u_{1}={\left(\frac{\sigma \mathrm{SGB}\lambda }{\rho }\right)}^{1/2}$$

In a strong SMF, the TEMF balances the MD force, i.e., $\sigma \mathrm{SGB}\approx \sigma u_{2}B^{2}$. The melt velocity can be derived as

(3) $$u_{2}=\frac{\mathrm{SG}}{B}$$

At the microscopic scale, the governing force balances are different.

In a weak SMF, the TEMF balances viscous friction, giving $\rho \nu u_{3}/\lambda^{2}\approx \sigma \mathrm{SGB}$. It leads to the melt velocity expression:

(4) $$u_{3}=\frac{\sigma \mathrm{SGB}\lambda^{2}}{\rho \nu }$$

while the flow velocity in a strong magnetic field still remains *u*_2_. Since *u*_1_ = *u*_2_ and *u*_3_ = *u*_2_, the critical maximum magnetic field intensity *B_max_* can be determined for macro- and microscales as

(5) $$B_{\max}^{\mathrm{Macro}}={\left(\frac{\rho \mathrm{SG}}{\lambda \sigma }\right)}^{1/3}$$

(6) $$B_{\max}^{\mathrm{Micro}}=\frac{1}{\lambda }{\left(\frac{\rho \nu }{\sigma }\right)}^{1/2}$$

Similarly, the corresponding maximum melt velocity *u_max_* is

(7) $$u_{\max}^{\mathrm{Macro}}={\left(\frac{\lambda \sigma }{\rho }{\left(\mathrm{SG}\right)}^{2}\right)}^{1/3}$$

(8) $$u_{\max}^{\mathrm{Micro}}=\mathrm{SG}\lambda {\left(\frac{\sigma }{\rho \nu }\right)}^{1/2}$$

where the parameters in the above equations are defined as follows: *S* is the Seebeck coefficient, *σ* is the electrical conductivity of the liquid phase, *ν* is the kinematic viscosity, *G* is the temperature gradient, *λ* is the characteristic length scale, *B* is the magnetic field intensity, and *ρ* is the density of the liquid phase.

Based on the derived equations, the variation in the melt flow velocity under different SMF intensities can be plotted. At weak SMF intensities, an increase in TEMC velocity can be observed with increasing SMF intensity, which means that TEMC is prevailing. However, when the magnetic field exceeds a critical value, a gradual decrease in flow velocity will be found at strong SMF intensities, indicating that the MD becomes dominant.

### 2.3. Magnetic Orientation

Early studies on orientation in the SMF were primarily conducted in the field of metallic materials. In 1981, Savitsky et al. [33] investigated the solidification of Bi-Mn alloys in a magnetic field of 2.5 T and discovered that the Mn-Bi phase aligned along the magnetic field direction, and the magnetization and magnetic permeability exhibited anisotropic characteristics. At the same time, Mikelson et al. [34] found that the precipitates oriented in directionally solidified Al-10wt.%Ni, Cd-60wt.%Zn, Al-35wt.%Cu and Bi-Cd alloys, for which they proposed the mechanism of rotational orientation. From then on, much work regarding orientation in different alloys in the SMF has been conducted [35,36,37,38,39]. Table 1 summarizes the magnetic alignment of different materials in the SMF. To explain these orientation phenomena, Asai et al. [6] analyzed the orientation mechanism based on the magnetization theory. They pointed out that magnetic orientation occurs only if the following three conditions are met: (1) The crystal must exhibit magnetic anisotropy. (2) The magnetic anisotropy energy must exceed the thermodynamic energy of the crystal. (3) The constraint forces from the surrounding medium must be weak enough to allow the magnetic force to rotate the crystal.

It was shown that the magnetization energy (*E_mag_*) of a crystal is closely related to its magnetization direction [47]. When the preferred orientation of a crystal is not parallel to the direction of the applied magnetic field, the crystal experiences a magnetic torque (*T*(*θ*, *B*)), which induces its rotation. The magnetic torque can be expressed as [48]

(9) $$T\left(\theta ,B\right)=-\frac{\partial E_{\mathrm{mag}}}{\partial \theta }$$

with *θ* the angle between the preferred crystal orientation and the direction of applied magnetic field. The magnetic anisotropy of crystals can be classified into two types: (1) Magnetocrystalline anisotropy, which arises from variations in magnetic susceptibility along different crystallographic directions. (2) Shape anisotropy, which results from geometric asymmetry in different crystal directions. The magnetic torques induced by these anisotropies, namely magnetocrystalline anisotropy torque (*T_mag_*) and shape anisotropy torque (*T_shap_*), can be expressed as follows [47]:

(10) $$T_{\mathrm{mag}}\left(\theta ,B\right)=\frac{1}{2}\chi \frac{B^{2}}{\mu_{0}}\sin(2\theta )$$

(11) $$T_{\mathrm{shap}}\left(\theta ,B\right)=\frac{(1-3n)}{4}\chi^{2}\frac{B^{2}}{\mu_{0}}V\sin(2\theta )$$

Here, *χ* represents the magnetic susceptibility, *μ*_0_ is the permeability of the vacuum, *n* denotes the shape anisotropy factor, *V* is the crystal volume. The magnetocrystalline anisotropy torque prefers aligning the crystal orientation with the highest magnetic susceptibility along the applied SMF direction. In contrast, the shape anisotropy torque favors the alignment of the crystal’s longest growth dimension with the SMF direction [47]. The effect of crystal orientation in the SMF can be utilized to prepare textured materials. This approach enhances the unique properties of crystals along specific directions, thereby improving the physical or mechanical performance of the material.

During the solidification of alloys, such as Al-Fe [49], Al-Sr [50] and Zn-Sn [51] alloys, the primary phase precipitates and remains surrounded by the liquid phase, providing a freely rotating medium. When the crystal exhibits significant magnetocrystalline anisotropy and possesses a sufficiently large size, it will undergo preferential orientation in an SMF to decrease its magnetization energy. If the eutectic phase continues to grow with the primary phase (like Zn-Mg alloy [52,53]), both the primary phase and the eutectic phase will simultaneously develop a preferential orientation under the influence of an SMF.

### 2.4. Magnetic Field-Controlled Diffusion

**Figure 3:**
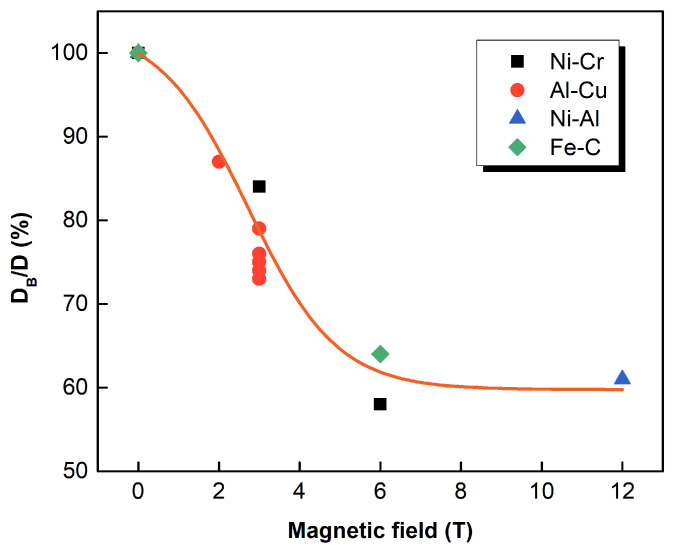
The ratio of the solid diffusion coefficient (*D_B_*/*D*) with and without an SMF in different alloy systems [8,9,55,57].

Back diffusion plays a key role in the formation of microsegregation during alloys’ solidification. Therefore, the change in diffusivity in the SMF also influences solidification behavior. It was shown that solid-state diffusion in the magnetic field exhibits certain variations. In 1964, Youdelis et al. [8,54] investigated the solid-state diffusion behavior of Al/Al-3wt.%Cu diffusion couples by applying an SMF of 3 T. They found that the Cu atoms’ diffusivity in the α-Al matrix was suppressed, and the solid-state diffusion coefficient decreased by approximately 25% when the SMF direction was parallel to the diffusion interface. However, the effects of SMF on the frequency factor *D*_0_ and the activation energy *Q* were not addressed. Further, they proposed the ambipolar diffusion theory and concluded that the suppression in the solid-state diffusion coefficient in the SMF was primarily due to a reduction in the *D*_0_ rather than a change in *Q*. For ferromagnetic alloys, Nakamichi et al. [55] detected that the diffusion of C atoms within the γ-Fe matrix was suppressed under an SMF of 6 T, whereas the solid diffusion of titanium (Ti) in the γ-Fe matrix was almost unaffected. They attributed this phenomenon to magnetic ordering-induced lattice stiffening. It gave rise to an increase in the migration activation energy of C and consequently reduced the solid-state diffusion rate. Similarly, Wang et al. [56] investigated the Fe-C diffusion system (Fe-0.76%C) in a 12 T SMF. Their results indicated that when the diffusion direction was vertical to the SMF, solid-state diffusion was significantly hindered, whereas diffusion was enhanced when the diffusion direction was parallel to the SMF. This was mainly because the austenite Gibbs free energy varied with C concentrations in the SMF. Additionally, the interaction between Fe atomic magnetic dipoles in the presence of the SMF resulted in diffusion coefficient anisotropy for C atoms. Yuan et al. [9] examined the diffusion behavior of Al in Ni via annealing Ni/Ni-6.3at.%Al diffusion couples with and without an SMF of 12 T. Their results showed that the SMF suppressed the diffusion of Al atoms in the direction parallel to the magnetic field, and the frequency factor *D*_0_ decreased by several orders of magnitude, while the activation energy *Q* remained almost unchanged. This provided further validation of the ambipolar diffusion theory proposed by Youdelis. Figure 3 compares the ratio (*D_B_*/*D*) of diffusion coefficients with and without an SMF in Ni-Cr [57], Ni-Al [9], Al-Cu [8] and Fe-C [55] alloy. It is found that the values of *D_B_*/*D* in different alloy systems show a similar tendency. The value of *D_B_*/*D* decreases with increasing SMF intensity until 6 T and tends to a constant when the SMF intensity goes higher. The behavior of solid diffusion in various alloys in the SMF is possibly consistent, which still needs tremendous experimental validation in the future.

Additionally, the reaction diffusion in an SMF has been deeply investigated as well. For instance, the interdiffusion between the Cu/Al solid–liquid interface under different SMF intensities was found related to the combined effects of MD and TEMC. The thickness of the diffusion layer of Cu/Al reached its maximum at 8.8 T and then decreased until 12 T [58]. Li et al. [59] also studied the reaction diffusion behavior of Cu/Al diffusion couples in the SMF and found that the diffusion layer thickness of diffusion parallel to the SMF was consistently greater compared than that perpendicular to the SMF. Furthermore, a gradient SMF inhibited the growth of intermetallic compounds, primarily due to the influence of vertical magnetic forces. Li et al. [60] employed an electroplating method to fabricate Ni-Al reaction diffusion couples and investigated their diffusion behavior in a an SMF of 6 T. Their findings revealed that the growth rate of the Ni_2_Al_3_ diffusion layer followed a parabolic relationship and the growth rate of the Ni_2_Al_3_ diffusion layer was sensitive to the direction between diffusion and the SMF.

The earliest theoretical study on the mechanism of the solid-state diffusion in an SMF was conducted by Youdelis et al. [54] in 1970. Based on plasma magnetohydrodynamics theory, they derived the following mathematical relationship for the solid diffusion coefficient with and without the SMF:

(12) $$D_{B}=\frac{D}{\left(1+\frac{\mu_{i}}{\mu_{e}}\cdot \frac{\omega_{\mathrm{ee}}^{2}}{\nu_{e}^{2}}\right)}$$

(13) $$\omega_{\mathrm{ee}}=\frac{\mathrm{eB}}{cm^{*}}$$

In this equation, *D_B_* and *D* represent the solid-state diffusion coefficients in the presence and absence of the SMF, respectively. The parameters $\mu_{i}$ and $\mu_{e}$ denote the mobility of ions and electrons, while $\omega_{\mathrm{ee}}$ and $\nu_{e}$ correspond to the electron cyclotron frequency and collision frequency, respectively. Additionally, *e* represents the elementary charge, $m^{*}$ is the electron effective mass, and *c* is the light speed. When $\omega_{\mathrm{ee}}^{2}/\nu_{e}^{2}\to \mu_{e}/\mu_{i}$, the solute atoms’ diffusion in solid is significantly suppressed by the SMF. However, when $\omega_{\mathrm{ee}}^{2}/\nu_{e}^{2}\gg \mu_{e}/\mu_{i}$, the ambipolar diffusion process is primarily controlled by electron motion. In this case, the rapidly diffusing electrons generate an electric field in space, which subsequently attracts ions, thereby altering their migration rate (i.e., diffusion rate). However, the calculations and experimental results obtained by Youdelis for the Al-Cu alloy did not match. They speculated that this discrepancy might be due to the perturbation effect of diffusing ions on the electronic band structure, which could affect both the electron collision frequency $\nu_{e}$ and the effective electron mass $m^{*}$. Nevertheless, no further analysis was conducted to verify this hypothesis.

Generally, the diffusion of Cu atoms in the Al matrix primarily occurs through the atom–vacancy jump mechanism [9]:

(14) $$D=a^{2}v\exp(\frac{∆S_{f}+∆S_{m}}{R})\exp(-\frac{∆H_{f}+∆H_{m}}{\mathrm{RT}})$$

where *a* represents the distance of the atom jump, *v* is the frequency of atom vibration, and ∆*H* and ∆*S* denote the changes in enthalpy and entropy, respectively. The subscripts “*f*” together with “*m*” correspond to the formation and migration of vacancies. By incorporating the form of the Arrhenius equation $D=D_{0}\exp(-Q/R\cdot T)$, $D_{0}$ and *Q* can be given as

(15) $$D_{0}=a^{2}v\exp(\frac{∆S_{f}+∆S_{m}}{R})$$

(16) $$Q=∆H_{f}+∆H_{m}$$

Experimental data indicate that the $D_{0}$ is changed in an SMF, while the *Q* keeps still. In a crystalline structure, the atomic jump distance remains constant for each jump [61]. Therefore, it can be inferred that an SMF may reduce either the atomic vibration frequency or the entropy change. However, the impact of the magnetic field on these two parameters remains unknown. Thus, the underlying mechanism of magnetic field-governed diffusion in the solid phase requires further exploring.

## 3. Undercooling in SMF

Undercooling is one of the important parameters influencing solidification. It was found that the nucleation temperature and undercooling of metallic melts would be modified by applying an SMF, indicating that the nucleation of metal melts is affected by the SMF [62,63,64,65,66]. Table 2 summarizes experimental measurements regarding undercooling alterations for various metallic systems in the presence of an SMF.

**Figure 4:**
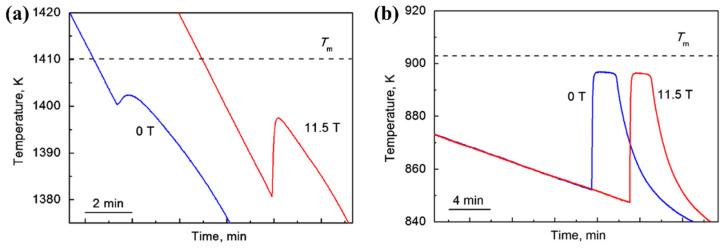
Cooling curves for (**a**) Ni-90wt.%Cu and (**b**) pure antimony in different SMFs [69].

As early as 1992, an enhancement in maximum undercooling in Cu melts was discovered in an SMF [67]. It was inferred that the MD effect of SMFs prevented thermal convection and consequently influenced the nucleation temperature. Similarly, the undercooling variations in pure Cu and Ge melts in the SMF were studied by Zhang et al. [68] using the glass fluxing method. Their results showed that as the magnetic field intensity increased, the average undercooling of Cu melts increased, whereas the undercooling of Ge melts exhibited no significant change. They attributed these phenomena to the magnetic braking effect, by which the convection vertical to the SMF was suppressed and the elimination of impurities and/or heterogeneous nuclei from the melt into the surrounding glass flux was promoted. This melt purification effect resulted in a larger undercooling for Cu. However, for Ge melts with a lower electrical conductivity, the magnetic braking effect was weaker and thus led to less effective convection suppression. Additionally, the high viscosity of Ge melts and the small density difference between Ge and its oxides hindered the removal of oxide impurities. All of the properties of Ge contribute to the negligible impact of the SMF on undercooling. Liu et al. [69] observed a reduction in nucleation temperature for both pure Sb and Ni-90wt%Cu alloy systems in the SMF, as shown in Figure 4. They attributed this phenomenon to the MD effect, by which the suppressed melt flow reduced the development of impurities and oxides that could act as nucleation sites.

**Table 2:** The modifications of undercooling in multiple metals and alloys with an SMF.

Materials	Magnetic Field Intensity	Degree of Undercooling	Ref.
Pure Aluminum	12 T	21.6 °C	[70]
	6 T	10 °C	[3]
Pure Antimony	6 T	9.6 °C	[4]
	11.5 T	5 °C	[69]
Pure Tin	6 T	7.6 °C	[4]
Pure Zinc	6 T	5.9 °C	[4]
Ni-90wt.%Cu	11.5 T	8 °C	[69]
Al-25at.%Cu	12 T	2.7 °C	[71]
Al-38wt.%Cu	12 T	10 °C	[72]
Al-4.5wt.%Cu	2 T	2 °C	[73]
	4 T	3 °C	[73]
	6 T	3.9 °C	[74]
	8 T	5 °C	[73]
Al-26wt.%Cu	6 T	7.3 °C	[74]
Al-45wt.%Cu	6 T	6.7 °C	[74]

Li et al. [75] developed a differential thermal analyzer capable of operating in strong magnetic field environments, by which they investigated the magnetic field-induced undercooling behavior of pure Al [3,70] and aluminum-based alloys with 3 wt.% nickel [76] and 25 at.% copper [71]. The fact that the undercooling of the pure Al, α-Al phase and CuAl_2_ phase increased as the SMF intensities rose was found. The modification of interfacial tension within the nuclei and melt induced by the SMF was mainly responsible for the altered nucleation process [3,70,76]. Additionally, they suggested that the remarkable suppressing effect of the SMF on the melt flow resulted in diffusion-controlled solute redistribution. Since the direction of atoms moving was opposite to the Lorentz force [71], their migration rate decreased, further influencing the nucleation process.

Wang et al. [77] conducted multiple measurements of the undercooling of pure Cu melt in the SMF using the glass molten salt encapsulation method. They observed that the undercooling gradually increased with increasing thermal cycles. When the SMF was applied at this stage, the undercooling increased significantly. However, after a certain number of thermal cycles, the undercooling reached saturation, and the application of the SMF no longer affected the undercooling. They suggested that convection helps to trigger nucleation by impurities and other heterogeneous nuclei. The MD effect effectively suppressed convection and reduced heterogeneous nucleation sites. As the thermal cycles increase, heterogeneous nuclei within the melt are gradually eliminated, which diminishes the influence of the MD on undercooling.

Cheng and Guo et al. [78] investigated the nucleation process when pure aluminum solidified on an Al_2_O_3_ substrate with a single-crystal structure in the SMF. Their findings indicated that the pure aluminum nucleation temperature decreased by approximately 2.9 °C in the presence of the SMF, leading to an enhancement in undercooling. A significant lattice misfit induced by the SMF between aluminum crystal and Al_2_O_3_ crystal near the interface was observed, to which the increasing interfacial free energy was attributed. Thereby, the formation of the critical nucleus and the undercooling behavior were modified.

In addition to melt convection and interfacial energy, researchers also attempted to interpret the mechanism of the nucleation in an SMF based on the interaction between magnetic dipoles. For example, Sun et al. [72] proposed the magnetic dipole interaction theory to explain this phenomenon. In this theory, the magnetized unit per unit volume (*V_u_*) acts as a single magnetic dipole. The mutual action between magnetic dipoles is determined by the angle formed by their moments and the unit vector. The magnetic dipole moment of magnetized unit *i* can be expressed as

(17) $$m_{i}=\frac{B}{\mu_{0}}\chi_{i}V_{u}$$

where $\mu_{0}$ and *χ* represent the permeability of the vacuum and the magnetic susceptibility of the magnetized unit. For the nucleation process at the interface, the interaction between magnetic dipoles can be expressed as

(18) $$F_{B}^{S}=-\frac{3\mu_{0}}{4\pi }\sum_{\mathrm{ij}}\frac{\left[\left(m_{i}\cdot m_{j}\right)-3(m_{i}\cdot e_{\mathrm{ij}})(m_{j}\cdot e_{\mathrm{ij}})\right]}{r_{\mathrm{ij}}^{4}}\propto \frac{3}{2\pi \mu_{0}}\sum_{\mathrm{ij}}\left(\frac{V_{u}^{2}}{r_{\mathrm{ij}}^{4}}\chi_{i}\chi_{j}B^{2}\right)$$

where $r_{\mathrm{ij}}$ is the distance between the two magnetic dipoles, $e_{ij}$ is the unit vector along the line connecting the centers of the two dipoles, and *i* and *j* represent the liquid-phase metal and the crucible at the interface, respectively. This interaction causes additional pressure at the interface, which leads to a change in the interfacial energy. The additional pressure can be expressed as

(19) $$P_{B}^{S}=\frac{-F_{B}^{S}}{S_{0}}\propto \frac{-1}{S_{0}}\frac{3}{2\pi \mu_{0}}\sum_{\mathrm{ij}}\left(\frac{V_{u}^{2}}{r_{\mathrm{ij}}^{4}}\chi_{i}\chi_{j}B^{2}\right)$$

where *S*_0_ is the interfacial area. $P_{B}^{S}$ is associated with the Maxwell magnetic pressure. It can also be written as $P_{B}^{S}=A_{ij}B^{2}/2\mu_{0}$. Thus, the magnetically driven interfacial tension would be obtained:

(20) $$\gamma_{B}=\frac{P_{B}^{S}}{2\kappa }≅\frac{A_{\mathrm{ij}}B^{2}}{4\kappa \mu_{0}}$$

where κ is the curvature, and $A_{ij}$ is the interfacial interaction factor related to the magnetic susceptibility of the magnetic dipoles. From above analysis, it can be demonstrated that the SMF will generate an additional interfacial tension. This, in turn, affects the wettability angle, further influencing the nucleation of the metallic melt. From this perspective, the magnetic dipole theory helps to qualitatively elucidate the modified undercooling in the SMF. However, the magnetic dipole theory still faces challenges. Quantitatively, the contribution of magnetic dipole interaction to the nucleation of the non-ferromagnetic metals remains quite limited and still remains to be validated by convincing experiments. Therefore, the underlying mechanism of nucleation in the SMF requires further investigation.

## 4. Interfacial Free Energy in SMFs

The solid–liquid interfacial free energy (SLIFE) is one of the most important fundamental thermodynamic parameters and affects nucleation and crystal growth during solidification [79,80,81]. Its magnitude and anisotropy are significantly correlated with interface stability, interfacial structure and phase transformation behavior [82,83]. Consequently, accurate experimental measurements for the SLIFE are necessary for understanding crystal growth and solidification phenomena. It also serves as a key input for the validation of experimental observations and the development of numerical models [84,85,86,87]. However, due to the inherent experimental challenges in external physical fields, studies on the influence of the SMF on the SLIFE in opaque metallic systems remain limited.

As an important interfacial parameter, the contact angle serves as a direct indicator of variations in solid–liquid interfacial energy. Li et al. [88] first measured the contact angle of Ga-In-Sn alloys in an SMF. It was revealed that the contact angle increased with the presence of an SMF. As a classical experimental approach for studying heterogeneous nucleation, the sessile drop method has been employed by Wang and Liu et al. [89,90,91] to investigate the contact angles of various metallic and substrate materials in the SMF. Their results demonstrated significant changes in contact angle. The possible reasons may come from the inhibited fluid flow under the MD effect and/or the modification of the solid–liquid boundary properties.

**Figure 5:**
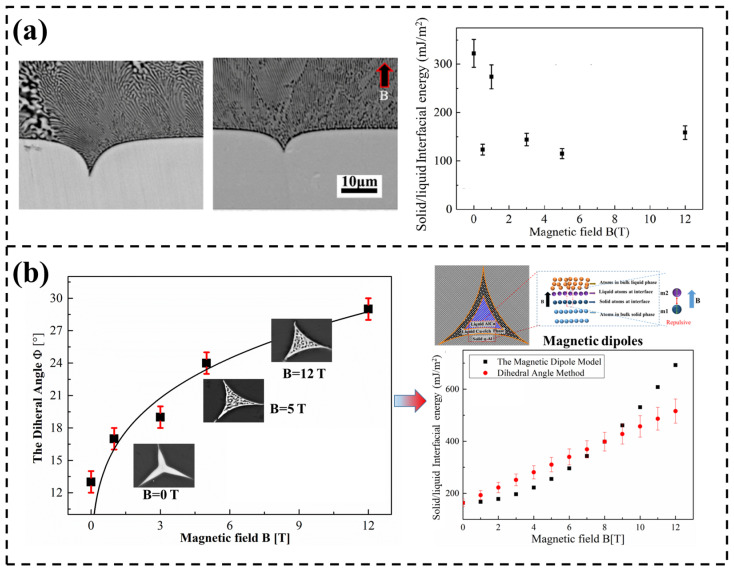
The effect of SMF on SLIFE measured by the IGBG technique (**a**) and DAM (**b**) [93,94].

Recently, an improved grain boundary groove (IGBG) method was employed to quantificationally assess the SLIFE in binary alloy (Al-Cu) in an SMF [92]. At a normal condition of 0 T, the interfacial free energy of the α-Al solid phase and the melt was 0.157 J/m^2^. It suddenly increased to 0.206 J/m^2^ when the SMF went to 5 T, corresponding to a 31.5% enhancement. For the interfacial free energy of the CuAl_2_ solid phase and the melt, it deceased from 0.280 J/m^2^ at 0 T to 0.151 J/m^2^ at 5 T, corresponding to a 46.2% reduction. More recently, Huang et al. [93,94] systematically validated the variation tendency of SLIFE in an SMF through the dihedral angle method (DAM) and the IGBG method. The relevant measurements of the two different technologies are shown in Figure 5. It can be seen that the SLIFE was quantified based on the observed solidification microstructures of grain boundary grooves (Figure 5a) and dihedral angles (Figure 5b). Experimental results revealed a threefold reduction in SLIFE in the applied SMF in both measurement approaches. Specifically, in the IGBG method, the pronounced reduction in the interfacial free energy in the SMF of 0.5 T was primarily attributed to TEMC at the sample scale. In contrast, a slight increase in interfacial energy was observed in a higher magnetic field intensity of 12 T, which is presumed to result from TEMC at the grain boundary groove scale and TEMF acting within the solid phase. The reduction in intrinsic SLIFE was further interpreted using the model of magnetic dipoles, as well as the structural ordering in the melt. These studies resolve previously conflicting and ambiguous observations regarding magnetic field-induced variations in SLIFE and provide compelling evidence that the interfacial thermodynamics during solidification could be modulated by an SMF. It should be emphasized that experimental measurements of SLIFE in the SMF for other binary or multi-component alloy systems need to be conducted in the future. Meanwhile, it is necessary to establish theoretical models of SLIFE in the SMF and to simulate the atomic arrangement, as well as energy distribution, near the interface, which is the one of the topics. This will be helpful to comprehensively understand the interfacial behavior in the SMF.

## 5. Microsegregation in SMF

Lots of studies have shown that the SMF can modify the microsegregation behavior during alloys’ solidification. This section systematically reviews studies on microsegregation in the SMF, highlighting existing controversies and challenges to provide valuable insights for researchers in this field.

As early as 1966, Chedzey [20] and Utech [21] simultaneously discovered that the application of the SMF during semiconductor crystal growth eliminated the band segregation caused by thermal fluctuations. They attributed this phenomenon primarily to the MD. Witt et al. [95] further applied a transverse magnetic field on the single-crystal growth process of Te-doped In-Sb alloy. It followed that the transverse magnetic field enhanced the melt viscosity, and therefore the thermal fluctuations caused by natural convection were stabilized. Additionally, a greater melt viscosity resulted in a larger vertical temperature gradient along the crystal growth direction, which helped prevent compositional undercooling and facilitated the growth of multi-component single crystals. For binary alloys, the Al-Cu system widely serves as typical model alloys. A 3.4 T transverse SMF was imposed on the unidirectional solidification process of a series of Al-Cu binary alloys to examine its effect on microsegregation [96]. It was revealed that the SMF was effective in reducing the effective partition coefficient and promoting positive segregation at pulling rates exceeding 3.5 μm/s. They speculated that this effect was due to an elevation in the liquidus in the SMF. However, when the Cu content was 0.5%, 4.5% and 7%, the effective partition coefficient showed an increasing tendency in the SMF, and therefore the extent of microsegregation reduced. The braked melt flow, as well as solid-state diffusivity, have been proposed to account for this phenomenon. Li et al. [97] further investigated the microsegregation behavior of Al-based binary alloys during directional solidification in SMFs. Their findings showed that the SMF reduced the grain boundary area, promoted grain coalescence, and increased solute solubility. This was attributed to the TEMF acting on the solid phase, which resulted in dendrite deformation and thus dislocation multiplication, and TEMF in the liquid phase, which promoted solute homogenization in the mushy zone. However, for equiaxed solidified Al-Cu alloys, they did not observe significant compositional segregation changes in the SMF. Shen et al. [98] exerted an SMF whose direction was perpendicular to the unidirectional solidification direction of Sn-Bi alloys and discovered that TEMC altered the solute distribution in the horizontal direction, ultimately refining the primary dendrite spacing. Yuan et al. [99] discovered that the Lorentz force generated by the application of an SMF hindered the migration of solute atoms from the solid–liquid interface into the liquid phase, thereby increased the solute content within α-Al grains during the solidification of Al-Cu alloys. Similarly, Song et al. [100] found that the SMF increased the maximum solubility of C in the α-Fe phase (ferrite) in Fe-0.027wt.%C alloys, suggesting that the phase transformation from the γ-Fe (austenite) to α-Fe phase (ferrite) would be accelerated considerably by the SMF.

**Figure 6:**
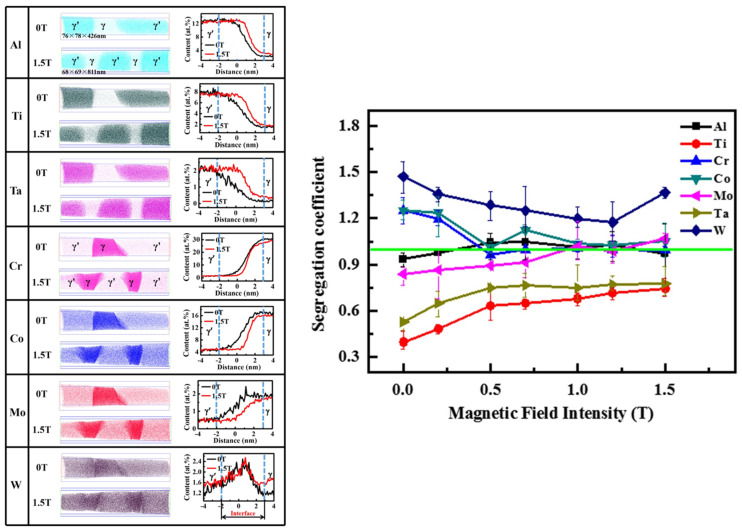
Atom probe analysis of solute content in γ/γ′ phase of unidirectionally solidified PWA1483 Ni-based superalloy (single crystal) in different SMFs and variation in segregation coefficient of each element [107].

For more complex multicomponent and multiphase alloys, the SMF also influences the solute distribution [101,102,103,104]. Hou et al. [105] examined the microstructure and microsegregation behavior of unidirectionally solidified Ni_54_Mn_24_Ga_22_ and Ni_48_Mn_30_Ga_22_ ternary alloys in a longitudinal SMF. Their observation demonstrated that the SMF refined the cell/dendrite structures and reduced the degree of microsegregation of alloying elements. Numerical simulations of liquid-phase flow revealed that the TEMC within the microscale (such as cells and dendrites) was the main factor. Ren et al. [106] clarified the influence of a strong SMF on the microsegregation of directionally solidified nickel-based superalloys. They observed that the SMF significantly reduced the dimension of γ′ precipitates, decreased the amount of carbides and γ/γ′ eutectic phases and consequently minimized microsegregation. In their latest study [107], they further found that the solute redistribution of γ/γ′ phases was changed by the SMF, as shown in Figure 6. The decrease in microsegregation led to a reduction in lattice misfit and influenced the precipitation process, ultimately significantly enhancing the creep life of single-crystal superalloy. Similarly, the degrees of microsegregation of Ti and Mo in DZ417G superalloys were reduced by 28% and 40%, respectively, in an SMF of 6 T [108].

Microsegregation behavior during rapid solidification—such as that occurring in welding and additive manufacturing—has also been found to be affected by the presence of an SMF. Chen [109] applied the SMF to Al/Ti laser welding. The forced convection in the melt pool owing to TEMC accelerated the transportation of heat and mass, thereby the microsegregation and the content of intermetallic compounds (TiAl_3_) decreased. Specifically, the bond strength of the Al/Ti welded joint was enhanced by 44.4% with an SMF of 120 mT.

The effect of the SMF on microsegregation has been analyzed from the point of view of MD and TEMC. Researchers generally attribute these phenomena to the influence of the SMF on the melt flow dynamic or phase transition temperature. Although these explanations are reasonable to some extent, they remain qualitative hypotheses, lacking direct experimental validation and quantitative analysis. Consequently, a theoretical model of microsegregation in the SMF needs to be established to accurately predict the solute distribution during alloys’ solidification. To overcome this limitation, an effective approach is to refine classical microsegregation models by systematically investigating the effect of the SMF on solidification parameters. Understanding how solidification parameters evolve in the SMF is crucial for constructing a comprehensive microsegregation model, which can provide guidance for the solute distribution during alloys’ solidification in the SMF.

The author’s team has conducted extensive work on establishing a theoretical microsegregation model in the SMF. Through a series of solidification experiments on alloys such as Al-Cu [73,110,111,112], Ni-Cr [57] and Ga-In [113] in the SMF, we have experimentally measured microsegregation and solidification parameters in the SMF, including undercooling, melt flow velocity, solid-state diffusion coefficient and dendrite coarsening coefficient. By establishing the correlation among these parameters with the SMF intensity and integrating classical microsegregation models, such as the Brody–Flemings [114] and Voller [115] models, we have ultimately developed theoretical microsegregation models that can be applied to calculate the extent of microsegregation in different SMFs [73,110].

In the case of directional solidification, experimental studies on microsegregation in Al–Cu alloys showed that an increasing SMF intensity led to enhanced microsegregation during planar interface growth [110,116]. This was because the SMF inhibited the solid back diffusion. In contrast, for cellular or dendritic growth, TEMC within the mushy zone induces secondary flows, which increase the effective partition coefficient and thereby reduce the degree of microsegregation. Thus, the variation in microsegregation in different solidification conditions is governed by the combined effects of retarded solid back diffusion and the TEMC in the melt. The Brody–Flemings model coupling with solid back diffusion can be adapted to incorporate the influence of the SMF on diffusion behavior. Given the presence of TEMFs and various convection effects during directional solidification in SMFs, the model can be modified by adjusting the partition coefficient to capture the effect of the SMF on microsegregation. Accordingly, based on experimental observation, the solid diffusion coefficient *D_s_* and the effective partition coefficient *k_e_* are revised within the Brody–Flemings framework to predict the extent of microsegregation in different SMFs. The modified Brody–Flemings equation in the SMF is expressed as follows:

(21) $$\frac{C_{s}}{C_{0}}=(a_{1}B+a_{2}){\left[1-\frac{f_{s}}{1+(a_{1}B+a_{2})\frac{4D_{B}t_{f}}{\lambda_{2}^{2}}}\right]}^{(a_{1}B+a_{2})-1}$$

(22) $$D_{B}=D_{s}\frac{10}{9.9+1.1B}$$

where *C_s_* is the solid concentration, *C*_0_ is the initial concentration, *f_s_* is the solid fraction, *a*_1_ and *a*_2_ are the coefficients related to solidification, *D_B_* is the solid diffusion coefficient under the magnetic field, *λ*_2_ is the characteristic scale, and *t_f_* represents the local solidification time.

In the case of equiaxed solidification, experimental investigations in Al–Cu alloys showed that the volumetric fraction of nonequilibrium eutectic displayed a gradual increase under the SMF, while the solute content in the primary α-Al phase decreased, indicating a deterioration in microsegregation [73,111,116]. The analysis suggested that the suppression of solid-state diffusion and the enhanced dendrite coarsening were both unfavorable for improving microsegregation. In contrast, the impact of magnetic field-induced changes in melt flow on the effective partition coefficient is relatively limited. Thermal analysis for the equiaxed solidification indicates that the application of the SMF increases the nucleation undercooling of the primary α-Al phase. However, since the undercooling variation is minor compared to that in zero-field conditions, its effect on microsegregation is negligible. Given that the growth of equiaxed α-Al crystals involves dendrite coarsening, which is significantly influenced by the SMFs, a modified microsegregation model for equiaxed solidification in SMFs was developed. This model is based on the Voller model and incorporates the effect of secondary dendrite arm coarsening. The governing equation of the modified Voller model in the SMF is given as

(23) $$\frac{4D_{B}t_{f}}{{\left(Mt_{f}^{1/3}\right)}^{2}}\frac{\partial C_{s}}{\partial \eta_{s}}+\left(k_{0}-1\right)C_{L}\frac{d\eta_{s}}{d\tau }+\left(\eta_{0}-\eta_{s}\right)\frac{dC_{L}}{d\tau }+\left(C_{L}-C_{0}\right)\frac{d\eta_{0}}{d\tau }=0$$

(24) $$\left\{\begin{array}{c} M={\left(-\frac{128{\sigma T}_{m}D_{\mathrm{eff}}}{∆Hm_{L}(1-k_{0})}\frac{\ln(C_{e}/C_{0})}{\left(C_{e}-C_{0}\right)}\right)}^{1/3}\\ u=BG\lambda^{2}\left(S_{S}-S_{L}\right)\frac{\sigma_{S}\sigma_{L}}{\rho \nu (\sigma_{S}-\sigma_{L})}\\ D_{\mathrm{eff}}=D_{\mathrm{in}}(1+\frac{1}{4}\lambda_{2}^{2}\frac{u^{2}}{D_{\mathrm{in}}^{2}}) \end{array}\right.$$

where *C_L_* is the liquid concentration; *η* and *τ* are dimensionless spatial variables and dimensionless temporal variables, respectively; *M* is the dendritic coarsening coefficient in the SMF; *D_eff_* is the effective liquid diffusion coefficient; *u* is the flow velocity; *D_in_* is the intrinsic liquid diffusion coefficient; *σ* is the interfacial free energy; *T_m_* is the melting temperature; Δ*H* is the latent heat of fusion; *m_L_* is the slope of the liquidus; and *C_e_* is the eutectic composition.

The models can predict the trend of the change in microsegregation during solidification in the SMF. However, there are still minor discrepancies between the predicted microsegregation from the model and the experimentally measured results. This deviation may arise from the following factors: (1) During the actual solidification of alloys, solidification parameters such as the coefficients of solid-state diffusion and dendritic coarsening vary with time or temperature. However, due to models’ limits, these parameters are assigned invariant values in calculations for simplicity. (2) The thermodynamic calculations of the alloy phase diagram are not coupled into the computational model, and the solidus and liquidus lines are simply assumed to be linear, leading to inaccuracies in certain parameters. (3) Measurement errors exist in determining the volumetric fraction of nonequilibrium eutectic.

The investigation of microsegregation in the SMF is crucial for the optimization of alloys’ development and casting processes. The establishment of microsegregation models in the SMF effectively guides the control of solidification structures and solute distribution homogenization. These research findings provide new insights and approaches for the development of advanced metallic materials. However, due to the complexity of multiple magnetic effects, many phenomena during alloy solidification in the SMF remain to be clarified. Currently, most studies simplify the problem through experimental observation and theoretical assumptions to establish mathematical relationship between the SMF intensity and key factors (e.g., solid-state diffusion coefficient, dendrite coarsening coefficient, etc.). Therefore, more precise theoretical and experimental studies are needed, particularly in the following aspects: (1) The accuracy of theoretical models and correction parameters requires extensive experimental validation. (2) Current theoretical models consider the solidification process in the SMF as a steady-state process, whereas actual solidification is transient, with many solidification parameters varying with time. Future research should incorporate time-dependent effects. (3) The microsegregation of practical multi-component, multi-phase alloys (e.g., superalloys and steels) in the SMF is more complex and requires further in-depth investigation.

## 6. Microstructure Evolution in SMF

Numerous studies have demonstrated that the application of the SMF can lead to a significant changes in microstructure during solidification with the aid of various magnetic effects, such as TEMC and MD [117,118,119,120,121].

It was shown that TEMC changed the dendrite morphology. Gel’Fgat and Gorbunov [30] observed that TEMC disrupted the crystal shape during the Czochralski growth of InSb crystals. Moreau et al. [12] conducted directional solidification experiments on a Bi-40wt%Sn alloy in an SMF of 0.55 T and found that dendrites became more developed, with the appearance of a spotted microstructure. Similarly, distorted cellular arrays and channel segregation in the mushy zone also appeared in a Pb-Sn alloy at an external SMF with an intensity of 0.45 T [13]. Different alloy systems show various microstructural evolutions under the SMF. For Cu-60wt.%Ag alloy, the primary dendritic arm spacing presented a non-monotonic trend with a maximum, whereas for Al-10wt.%Cu alloy, the primary dendritic arm spacing consistently increased [28,122]. Meanwhile, the dendrite arrays became highly disordered, and spotted microstructures appeared in the mushy zone when the SMF was sufficiently high. The authors attributed these findings to the competition between thermal solute convection and TEMC.

**Figure 7:**
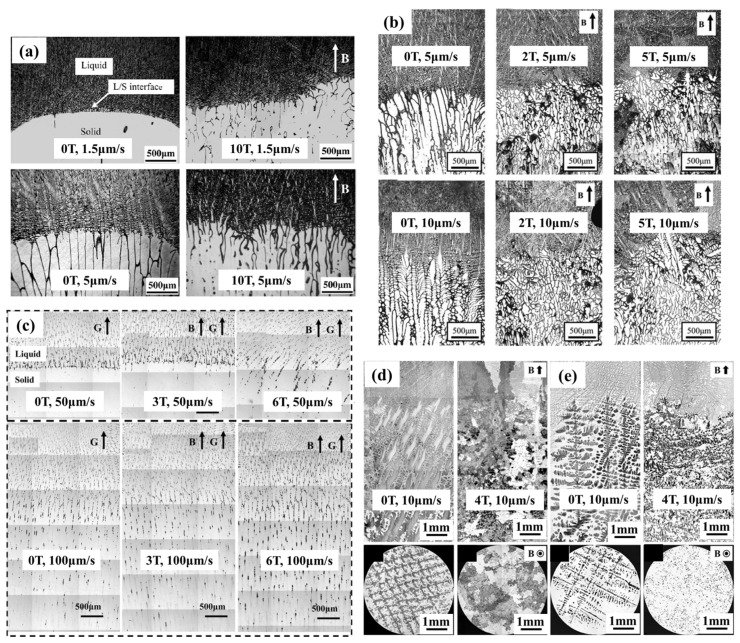
The quenched morphology of solid–liquid interfaces in different alloys in various SMF intensities with different directional solidification rates: (**a**) Al–0.85 wt.% Cu, (**b**) Al–4.5 wt.% Cu, (**c**) Ni-10wt.% Cr, (**d**) Ni-Al_21.5_-Zr_0.4_-B_0.1_ (at.%), (**e**) Ni-Al_26.5_-Zr_0.4_-B_0.1_ (at.%) [57,110,125,127].

Additionally, the solid–liquid interface morphology during the solidification process was demonstrated to be affected by the TEMC. Ren et al. [97,123,124] systematically conducted comprehensive studies on interfacial issues under the SMF in terms of interface stability, evolution of interface morphology and dendritic growth in Al-M (M = Cu, Si) hypoeutectic alloys. Their study revealed that TEMC was initially intensified and then weakened by increasing the SMF. Destabilization of the solid–liquid interface under the SMF was found [32,125,126]. Figure 7 shows the interfacial transition from planar to cellular and dendritic structures in various materials. When the alloy solidified with a cellular interface in a weak longitudinal magnetic field, a “tree ring-like” morphology was observed in the mushy zone, whereas in a weak transverse magnetic field, the cellular morphology remained unchanged. Furthermore, in a weak transverse magnetic field, secondary dendrite arms of columnar dendrites extended preferentially in a specific direction. The SMF also severely distorted cell/dendrite clusters, enhanced secondary branching and accelerated the growth of higher-order dendrites. These phenomena were attributed to the existence of TEMC near the solid–liquid interface, with a nonlinear response between TEMC velocity and the applied SMF. Moreover, the SMF influenced dendrite growth’s spatial alignment, leading to the formation of new preferential growth orientations. Both TEMC and MD were found to be scale-dependent, and their competition at different characteristic scales (crucible scale, primary dendrite scale, secondary dendrite scale) contributed to the unique variations in the solidification structure.

**Figure 8:**
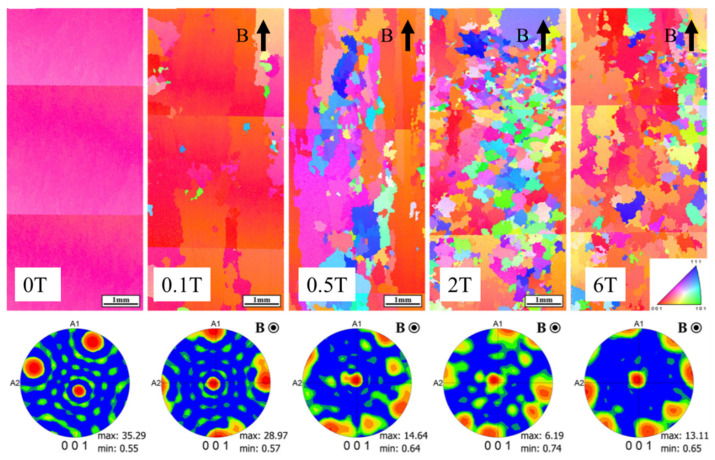
CET transformation of directionally solidified Ni-22Al alloy in axial magnetic field [138].

The SMF can induce columnar-to-equiaxed transition (CET) in the directional solidification of Al-Cu alloys, bearing steels and nickel-based superalloys, as shown in Figure 8. The critical magnetic field intensity for this transition was found to show a proportional dependence on the withdrawal rate and have a negative relationship with the temperature gradient [128,129,130,131]. During the growth of columnar dendrites, fragmented dendrite arms can act as nuclei for equiaxed grains [132].

The fact that the CET was significantly dominated by the fluid flow was proved by solidification experiments in a microgravity condition [133]. The flow facilitated the transport of dendrite fragments of Al-7wt.%Si alloy to the front of the dendritic array. Thereafter, the fragments started nucleating and continued to evolve into equiaxed dendrites in the undercooled melt, thereby hindering the growth of columnar dendrites. As discussed earlier, the SMF can alter melt flow during solidification, thereby affect CET. Spittle [134] observed that a fully equiaxed grain structure could be obtained under a 0.2 T SMF when the Sn-Pb alloy was directionally solidified by chill casting. To further clarify the underlying mechanisms, Li et al. [129] conducted in-depth studies about the effect of an SMF on the directional solidification of Al-4.5Cu, Pb-80Sn, DZ417 nickel-based superalloy, Al-15Cu and Al-40Cu alloys. In all cases, the primary phase exhibited CET, and the required SMF intensity for CET was proved to be proportional to the pulling rate. Additionally, the CET also happened in multiple alloy solidification (e.g., Sr-modified Al-7Si) at the condition of a transverse SMF [135]. Using synchrotron radiation techniques, the formation and migration of equiaxed grains during directional solidification was observed in situ [136,137]. These experiments provided direct evidence that TEMF induced the fragmentation of dendrite arms, acting as nuclei. The results demonstrated that TEMF played a crucial role in driving dendrite fragments’ movement, offering valuable insights into the long-standing challenge of dendrite fragmentation due to stress and its role in nucleation. Based on these findings, the SMF could be one of the potential approaches utilized to manipulate the morphology of columnar and/or equiaxed dendrites during directional solidification, opening novel approaches for the control of grain growth during solidification.

Based on the aforementioned results, TEMF and TEMC are considered to be a promising way to optimize the solidification microstructures of continuous casting billets. Hou et al. [131,139] illustrated the mechanism of the microstructure evolution of bearing steel when imposing an SMF. In directional solidification experiments with a temperature gradient of 104 K/cm and a pulling rate of 20 μm/s, the columnar dendritic network at the edges of the specimen was disrupted and became irregular with a 2 T SMF. When the SMF went to 5 T, the disordered area started to expand to the central region. These findings indicated that the columnar grain disorder was triggered by the SMF and finally induced the CET. They attributed the CET in the SMF to two factors: (1) The TEMF directly acting on secondary dendrites generates stress concentrated at the root. For secondary dendrites of typical sizes, this stress can reach the order of 10^−1^ MPa. According to the stress remelting mechanism, stress of this magnitude can cause dendrite root rupture within approximately 10^−1^ s, making it a primary factor in dendrite fragmentation. (2) The TEMC between primary dendrites can reach flow velocities on the order of 10^3^ μm/s, which is sufficient to drive dendrite fragments or free nuclei toward the sample’s edges and the columnar grain front, facilitating the CET process.

In parallel, the MD effect of the SMF has been extensively used to avoid the structure defects caused by heat oscillation and/or unstable flow in bulk crystal growth [140]. This concept was well developed by Hoshi from Japan in the 1980s [141]. The theory showed that the MD effect would diminish the transportation of impurities and extra liquid flow, and therefore a Si crystal with a high quality could be obtained. From then on, plenty of experimental and theoretical work has been conducted to enable the practical application of SMF technology in the crystal growth industry, containing Si crystals [142], GaAs [143,144,145], GaSn [146], InP [147], TiO_2_ [148], etc. It should be noticed that some defect-free crystal growth with a large dimension cannot be obtained simply through the rotation of the crucible and/or crystal itself. To achieve this, it must rely on the effect of the SMF [149]. Nevertheless, the most significant issue for SMF-assisted bulk crystal growth is an economical high-intensity superconducting magnet with a sufficiently large uniform intensity area. As an illustration, it is necessary for the Czochralski growth of Si that the magnetic field should be of sufficient intensity and that the effective region of the magnet must cover at least 1000 mm. Finally, it has been realized that the effects of SMFs on the melt flow and heat transfer are highly intricate within a dynamic nonlinear system. Thus, numerical simulation models were developed [150,151,152,153,154], which provide a good scientific basis for the use of SMFs.

Studies regarding microstructures in the SMF have been well performed both in calculation and experiments. The fundamentals are primarily established. Nevertheless, microstructure evolution in the SMF is dominated by various effects. Sometimes these effects are detrimental to the alloys’ performance. Thus, the major concern in the future is the balance of the different effects of the SMF to obtain well-controlled microstructures for practical application.

## 7. Summary and Prospective

EPM has evolved into a new interdisciplinary field over the past decades, and the SMF has become an essential technique for producing high-quality alloy castings. With the development of new alloy systems such as special steels, superalloys and high-entropy alloys, there is an increasing demand for improving their casting efficiency and casting quality, as well as the development of advanced solidification techniques. Up to now, the SMF has been found to affect nucleation, dendrite growth, microsegregation and microstructure, etc., during solidification. Thus, the SMF has become a promising method to tailor the microstructures and performance of the alloys. In particular, an ultra-high SMF exerts significant effects on material processing, even influencing atomic-scale behaviors. It is shown that ultra-high SMFs impact both thermodynamics and kinetics, opening up vast opportunities for their application in material processing. Nevertheless, the SMF might also bring detrimental effects that should be avoided. For example, the attenuated solid diffusion under an SMF in some alloys possibly deteriorates element uniformity. Additionally, the TEMC on macroscales is always beneficial to the formation of solute plumes, and finally these become segregation channels, which may induce macrosegregation during directionally solidified alloys. Additionally, EPM is expected to develop towards multi-mode, multi-functional, hybrid and customized magnetic field applications. With advancements in superconducting technology, the emergence of an ultra-high SMF can generate more economical, convenient and potentially revolutionary changes in material processing techniques. For industrial processes regarding magnetic effects, the following aspects should be paid attention to. The first question is how to economically construct magnets with a high intensity and large-size working space. Although superconducting magnet technology is well developed, it still faces challenges, i.e., the higher the magnetic field is, the smaller the working space of the magnet. Additionally, special equipment should be designed to match the magnet, such as directional solidification apparatus. Thirdly, since the thermodynamic/dynamic parameters of materials could be modified under the action of the SMF, these parameters need to be carefully measured, and thus new scientific instruments that can be used in the magnetic field need to be developed as well. Those aspects are the basis for industrial processes.

The solidification process is a complex, multi-physics coupling phenomenon involving mass/heat transportation and microstructure evolution. The SMF further complicates these interactions, making it crucial to accurately understand their relationships for the effective application of the SMF. To achieve this goal, the integration of artificial intelligence (AI) and big data analytics is indispensable. AI-driven modeling and optimization will provide a deeper understanding of the effect of the SMF on solidification dynamics, enabling more precise control over process parameters and microstructure evolution. As for AI technologies, their capabilities in terms of image capturing, analyzing and constructing could be helpful for the study of microstructure evolution under the SMF, cooperating with the in situ observation of synchronous radiation. The complicated mathematic issues of magnetohydrodynamics are expected to be easily solved by AI. This will be a key direction for the future development of EPM, paving the way for smarter, more efficient and adaptive material processing.

In summary, the practical implementation of the SMF in industry is still up against several technological challenges. (1) The preparation of large-size alloy ingots, such as continuous casting billets, cast turbine blades, etc., in the SMF is difficult owing to restrictions in the working space of SMF devices. (2) The synthetical effect of the SMF in macro- and microscales on liquid convection, microsegregation and interfacial behavior needs more in-depth investigations. (3) It is necessary to develop novel equipment to measure the change in parameters of materials in the SMF. (4) It is an open question as to the coupling effects of the SMF with other physical fields like electric fields and ultrasonic fields. The above issues should be paid especial attention in the next decades.

## Figures and Tables

**Table 1:** Summary of magnetic orientation of different alloys in SMF.

Substance	SMF Intensity	Orientation Relationship (z-Axis and *B*)	Ref.
NiAl_3_ (orthorhombic)	10 T	Parallel	[34,40]
BiMn*_x_* (hexagonal)	10 T	Parallel	[33]
CuAl_2_ (tetragonal)	10 T	Parallel	[41]
Zinc (hexagonal)	1 T	Parallel	[34]
Bismuth (hexagonal)	5~12 T	Perpendicular	[14]
SbMn (hexagonal)	12 T	Parallel	[42,43]
Co_17_Sm_2_ (hexagonal)	2.5 T	Parallel	[44]
FeAl_3_ (monoclinic)	12 T	<543> parallel to *B*	[45]
Nd_2_BFe_14_ (tetragonal)	5 T	Parallel	[46]

## Abbreviations

The following abbreviations are used in this manuscript:

SMF	Steady magnetic field
EPM	Electromagnetic Processing of Materials
MD	Magnetic damping
TEMC	Thermoelectric magnetic convection
MHD	Magnetohydrodynamics
TEC	Thermoelectric current
TEMF	Thermoelectric magnetic force
SLIFE	Solid–liquid interfacial free energy
IGBG	Improved grain boundary groove
DAM	Dihedral angle method
CET	Columnar-to-equiaxed transition
